# Supplementary material for: The role of doulas in abortion care in Canada: A qualitative study
Source: PLoS One. 2025 Mar 3;20(3):e0313918. doi: 10.1371/journal.pone.0313918 (PMC11875338; doi:10.1371/journal.pone.0313918)
Supplement: Attachment — Submitted filename: Responses to Reviewers 20240703 (1).docx [file pone.0313918.s002.docx]

Responses to Reviewers

| Reviewer #1: |  |  |
| --- | --- | --- |
| While I loved the content of the paper, I felt a little underwhelmed by the framing. The three themes (what do abortion doulas do; challenges to abortion doula work; facilitators to abortion doula work), just didn’t feel like themes that emerged from the data. These felt like they could have easily come from the aims of the project. I just would have rather it was noted that the paper was organized along these lines and not that these emerged through analysis. | Thank you for your thoughtful feedback. We have revisited our analysis and re-organized into six key themes: Inter and intra-systems navigation; After-care; In over their heads; Balancing act; Support for the supporter; and Beyond the individual. We feel that these themes speak more accurately to the work of abortion doulas represented in the data. |  |
| I just felt a bit underwhelmed by the sub themes | See comment above. |  |
| … I would say in general I felt like the paper could make more of a theoretical intervention - or at least draw on theory — more than it does. | Thank you, we have integrated reproductive justice theory into the framing and discussion of key themes. |  |
| For example, there were many references to stigma but no mention of Goffman or the many, many advances on Goffman’s theory in the reproductive health space. | We have added further background on abortion stigma on lines 106-113. |  |
| I was very surprised not to see mention of Reproductive Justice. While race and racism was mentioned briefly in the background, it wasn’t at all present in the paper. Was this not present in the data? | Thank you, we do work from a framework of reproductive justice, but this was not made explicit in the paper. We have added a section in the methods on how reproductive justice theory guided our study design and implementation. While doulas did not explicitly mention reproductive justice, we have commented throughout on where their work aligns with a vision of reproductive justice. |  |
| There was a mention of birth doulas as an intervention on racism — how is this considered in abortion doula work? (Again, this feels like it relates back to wanting more about if and how doulas intervene upon interpersonal and structural stigma) | We have added citations to explain that, like in perinatal care, racialized and queer patients may face structural barriers to abortion care. |  |
| I also thought Sustainability could have gone a bit further — the financial instability is such a huge challenge. I wanted to know more. The idea of putting a band aid on the problems — I just wanted to know more about that kind of meaning making, how the doulas saw their role and what it meant for them; how does this intersect with advocacy work? Are the doulas dealing with burn out from this? | We have collapsed sustainability into Theme 4: A balancing act. We feel this better communicates how the doulas saw their role and expressed the challenges regarding sustainability, burn-out, and the ethical dilemmas between providing equity-based care and seeking renumeration. Within this new theme, we have expanded on both the financial instability and the doulas perceptions of short-term fixes to structural inequalities in care. |  |
| What I would love to see is an expansion of the themes — say more than “complex” and “community” — and a digging into the meaning of these barriers and facilitators. I think it would make the paper more powerful and really has potential to expand the literature on doula work, reproductive justice, abortion, and stigma | Thank you, see comment above re: expansion of themes. Additionally, we collapsed several themes that were previously presented as either “barriers” or “facilitators” to more accurately reflect the nuanced nature of the abortion doula work, where healthcare systems and models of work can both facilitate and challenge the work. |  |
| **Reviewer #2:** |  |  |
| The authors note that they conducted the study in partnership with non-academic experts in doula care, which is a strength. Can they provide more details about how this partnership came about? Did it exist prior to the study? | We have added further detail on lines 161-167. |  |
| As noted, the authors provide a sensitive review of the various underserved groups who may face additional complexities when accessing abortion care. Did any members of the study team (including the non-academic partners) have lived expertise of any of the conditions or identities experienced by these groups? | Added detail on lines 168-9. |  |
| Roughly one fifth of the doulas recruited to participate in interviews agreed to do so, which is an adequate sample. However, it would be helpful to hear more about the recruitment efforts: what role, if any, did the non-academic experts play in designing the recruitment materials? How did the team attempt to make study participation appealing (beyond the honorarium, and including what information was provided in the recruitment message regarding confidentiality and assuring participant safety)? | Added detail on line 175-178. |  |
| It’s noted that the interviews were conducted on Zoom; how many interviewers were on each call? Did the non-academic partners co-facilitate any of the interviews? | Added detail on line 195-197. |  |
